# Gestures, Objects, and Spaces: Exploring Teachers’ Multimodal Communication in Nursery Schools

**DOI:** 10.5964/ejop.12291

**Published:** 2024-05-29

**Authors:** Ana Moreno-Núñez, Marta Casla

**Affiliations:** 1 Departamento Interfacultativo de Psicología Evolutiva y de la Educación, Universidad Autónoma de Madrid, Madrid, Spain; Concordia University, Montreal, Canada

**Keywords:** multimodality, circle time, early childhood education, gestures, materiality, space

## Abstract

This study builds on the increasing evidence that the multimodal nature of adult-child interactions and the use of objects play an important role in early linguistic development. Most of these studies analyzed dyadic interactions at home, whereas few research has been conducted in early childhood education and care settings. In this paper, we characterized the multimodal nature of teachers’ communicative bids during classroom-based group interactions in nursery schools. Observational data of circle-time activities was collected from 16 Spanish nursery school classrooms, comprising 16 teachers and 161 children between two and three years of age. We analyzed teachers’ communicative bids (i.e., verbal utterances and verbal-gestural bids) considering the frequency of use of different types of gestures, to whom are they addressed (i.e., the whole group or a single child), the extent to which they involve the use of objects, the classroom layout, and the relationship between the communicative bids and the number of children that participated in each classroom. Teachers’ communication with toddlers is highly multimodal and rely on different types of gestures, although the use of objects in our sample was scarce. Descriptive analysis suggest that certain classroom layouts may favor teachers’ use of some types of gestures over others. In this article, we discuss the implications of both the use of objects and space for understanding how adults shape the linguistic contexts of young children, and the potential opportunities and limitations they pose for classroom interactions.

Adults frequently interact with children according to organized and predictable instances that are built around materiality (Alessandroni, 2023), thus promoting relevant experiences and forms of behavior that are consistent with their cultural rules of use (Kärtner, 2018). During the first years of life, children’s early experiences with the material world are co-constructed through joint actions with others, in which they acquire an increasingly active role (Moreno-Núñez et al., 2017). These material encounters are not limited, though, to the manipulative exploration of objects, but must be considered from a broader perspective that also accounts for the features of the context in which the interaction takes place, including the space and its organization. While both space and materiality refer to complex concepts that even partially overlap, in early childhood education studies space typically refers to the physical environment of nursery schools, while materiality is understood as the physical qualities of objects and elements of nature (Løkken & Moser, 2012). Therefore, space, materiality and social interaction are entwined, as they all contribute to providing opportunities and limits for human interaction.

While educational contexts, such as nursery schools, have proven to be an important niche for the development of basic communication and social interaction skills, their operationalization as part of the children’s early linguistic context is still limited. There is ample evidence of how early communicative interactions between mothers and infants shape their linguistic developmental trajectories. However, only a few studies describe the interactions that take place outside the home context. Additionally, there is a clear imbalance in basic and applied research in favor of the 3- to 6-year-old period, while studies on early childhood education (i.e., under three years of age) are significantly scarce. Considering that early schooling is a reality for most Western children, it is worth exploring its role in ensuring opportunities for infants’ participation with other adults and peers (Pianta, 2016).

Depicting the educational reality of children should acknowledge how everyday communicative instances in the classroom unfold, for which ecological research may be particularly appropriate. To this goal, exploring how teachers’ pedagogical and communicative practices are geared through materiality would allow us to identify not only the resources they use and how they are structured, but also what kind of opportunities for participation they promote and what the factors that may influence them are (Bautista et al., 2020). The interrelationships between the adult's action and the children's responses could contribute to our understanding of how these inhibit or promote children’s participation, meaning-making and learning experiences. Hence, the overall purpose of our research is to investigate the relationship between materiality and teacher use of communicative behaviors (e.g., gestures) during group interactions in nursery school classrooms.

## Language Developmental Contexts and Early Childhood Education

Despite its voluntary nature, early access to schooling has increased in recent years due to new social and family realities. This has also been associated with a positive impact on children's development (García et al., 2020), e.g., in their social competence and engagement (Degotardi, 2021), especially when the education they receive is of high quality. Educational quality in early childhood education is usually assessed in terms of criteria such as a high level of teacher education and adequate resourcing for centers, that includes low adult-child ratios and small group sizes (Vermeer et al., 2016). Low-ratio settings have been typically associated with better opportunities for children to interact with adults, though a recent meta-analysis suggests that their effects on children’s developmental outcomes are the result of complex interactions between several variables, and that they might be compensated for larger ratio situations through, for instance, the teacher’s professional competence (Dalgaard et al., 2022).

In early childhood education, children's participation in their daily classroom routines has proven to be an educational tool with extraordinary potential for the acquisition of communicative competencies, as they get involved in their own educational process and the generation and development of interactive spaces (Justice et al., 2018). Over the last few decades, there has been a growth in research demonstrating the strong relationship between child-directed speech (CDS) and early language development (Rowe & Snow, 2020). In a similar way to mothers, teachers adjust the amount and complexity of their CDS according to the age and diverse linguistic abilities of a varied group children (Cárdenas et al., 2020; Degotardi, 2021; Justice et al., 2018). Indeed, recent research has stressed that each child’s particular linguistic experience at school may also quantitatively and qualitatively differ from that of their peers (Perry et al., 2018), even within the same group (Chaparro-Moreno et al., 2019). This dynamic nature of teachers’ speech also accounts for the distribution of their talk according to structural factors such as the classroom ratio (Degotardi et al., 2018) or whether the interactions take place during structured activities (Soderstrom & Wittebolle, 2013) such as circle time or shared reading. In fact, while children’s linguistic participation during these group activities does not exceed 16% of the time (Torr, 2019), they seem to contain higher concentrations of adults’ talk, as compared to other everyday school routines. Thus, interactive activities could lead to a more homogeneous experience for each child (Chaparro-Moreno et al., 2019), although the child’s linguistic growth could also be influenced by the group size and the language skills of his or her peers (Degotardi, 2021).

In addition to interactive group activities, the use of objects and spaces to convey communicative behaviors in early childhood is a powerful and effective pedagogical strategy, with the potential to help children to engage in collective communicative interactions with peers and adults. Regardless of who initiates the interactions, the way in which teachers guide the course of the dialogues provides of structure to everyday conversations and encourage children to make relationships with other ideas. However, the scarce knowledge about how children's linguistic context is shaped outside home settings (Degotardi, 2021) requires further exploration and should focus on how and under what circumstances the exchanges with other people and the material world unfold.

## Materiality and Multimodality in the Promotion of Interaction

The term materiality has been increasingly used when explaining the impact of things on human experience, actions, and communication. However, its complex nature brings the study of material and organizational structures of the early childhood classroom at the center of a multidisciplinary juncture that includes areas such as architecture, geography, and social and educational sciences (Løkken & Moser, 2012). This has broadened the focus on materiality by not only examining the use of objects and artifacts or their physical qualities, but also other elements such as the physical characteristics of the school context (for example, how the classrooms look, or the furniture and outdoor spaces available) and their spatial organization. Accordingly, ideas on materiality and spaces can therefore be seen as being constituted through the interaction between educators and children, situated within the educational and organizational framework of the activities in which they all participate. Importantly, material characteristics determine, to a greater or lesser extent, the possibilities of experimentation and participation of children and adults on the sensorimotor experience and the process of co-construction of meanings.

Given the importance of the material environment, it seems relevant to explore the role that materiality and spaces play in the educational practice of early childhood teachers, as this allows for a deeper knowledge about daily experiences in the classroom. Although even widespread pedagogical models, such as those based on the Montessori philosophy, emphasize the importance of the material and structural context in children’s development, there are relatively few studies that empirically explore its educational relevance and how it is articulated in the child's everyday classroom routines. We believe that studying the impact of these factors requires adopting a complex exploratory approach based on participation as a tool for social construction (Berge, 2019). In this sense, the different practices that take place in the school could help us to understand the foundations, materials and strategies on which educators rely to enhance interaction and communication in the school.

We should also note that increasing evidence supports multimodality as a key feature of the early communication system (Perniss, 2018). The coordination of multimodal actions has been shown to be an important interaction tool for both adults (Rowe & Goldin-Meadow, 2009) and children (Wu & Gros-Louis, 2014). In particular, mother-child interactions have largely informed the literature on communicative multimodality (Suanda et al., 2016), showing how verbal and gestural-coordinated utterances occupy an important place in facilitating and shaping children's communicative developmental trajectories (Rodrigo et al., 2006; Rowe & Goldin-Meadow, 2009; Tamis-LeMonda et al., 2012). Moreover, adults’ gestures directed to children are usually accompanied by verbal utterances and rarely occur alone (Iverson et al., 1999). Their frequent use of verbal-gestural bids also seems to drive children's multimodal behaviors (Rodrigo et al., 2006), as well as having a predictive value in their subsequent language skills (Rowe & Goldin-Meadow, 2009; Tamis-LeMonda et al., 2012).

Even though this is a widely observed trend, some studies highlight certain differences in adults' use of gestures that, consequently, indicate the diversity of linguistic contexts to which children are exposed. On the one hand, cultural factors underlying parenting practices could explain some of these differences since, for instance, Latino mothers tend to use more gestures when interacting with their children than mothers from African-American backgrounds (Tamis-LeMonda et al., 2012). On the other hand, the use of gestures may vary according to their type and whether or not they involve the use of objects. More specifically, deictic gestures, especially pointing, tend to be the most frequent gestures in mothers (Tamis-LeMonda et al., 2012) and, together with instrumental gestures, are significantly more frequently used to interact with their children at 12, 24 and 36 months than symbolic and social gestures (Rodrigo et al., 2006). However, since most of these studies have focused on mother-child interactions, their potential similarities and differences with children's linguistic experiences in school settings remains underexplored. One exception is the study conducted by White et al. (2015), showing that teachers’ use of gestures elicited more children’s responses than solely verbal utterances. Although this study did not distinguish among the types of gestures, teachers may use gestures differently to accompany their verbal utterances in order to convey diverse meanings adjusted to the characteristics of materiality.

Although variables such as classroom ratios, child age, and teacher professional development have been identified as some of those that might correlate with teacher language (Degotardi et al., 2018), there are still many unanswered questions about the language environment to which young children are exposed. For instance, it is important to analyze the extent to which objects are part of those interchanges, as well as within-group (Chaparro-Moreno et al., 2019) and between-group variations (Hindman et al., 2021) in the linguistic experiences of the children. The role of space in the use of different communicative behaviors is especially interesting in nursery schools, where teachers simultaneously interact with children whose linguistic abilities usually differ. For example, structured activities such as circle time might bring good examples of how teachers shape a meaningful learning space for every child, ensuring the provision of opportunities for communicative and social participation. To the best of our knowledge, research with observational studies focused on the school linguistic environment of non-English-speaking countries is very scarce (Degotardi, 2021). The potential variations in the interactions analyzed here could cast light on the role of teachers’ speech in early linguistic development.

## Aims

The overall purpose of our research was to explore the characteristics of teachers’ communicative bids during classroom-based group interactions in nursery schools. To this goal, we explored the quantitative and qualitative variations of teachers’ multimodal coordination based on (1) the frequency of use of gestures and their different types, (2) the extent to which they involve the use of objects and are related to the classroom layout, (3) to whom each communicative bid was addressed (i.e., the whole group or a single child) and, (4) its relations with the teacher-student ratio. We expected the number of children in the classroom could be related to the multimodal nature of teachers’ communicative bids, the use of objects and the frequency of group-directed utterances. We also expected specific configurations of gestures, the use of objects and the classroom layout to be coordinated with the verbal utterances directed to the children. In particular, we considered that some classroom layouts might favor greater opportunities for group-directed communicative bids, while other layouts might be more inviting to address children in one-to-one interactions.

## Method

### Participants

This study involved 16 female teachers in charge of 16 classrooms across six public nursery schools in middle-income urban areas of the Madrid region (Spain) and 161 children attending the 2–3 years classrooms. Nursery schools were selected from an intentional sampling based on previous contacts of the research team. Children’s mean age was 29.33 months (*SD* = 3.9 months). The number of children per classroom ranged from six to 15 (*M* = 10.06).

### Procedure

Prior to data collection, two researchers conducted a participatory observation in each classroom to familiarize the children with them and the recording equipment. They took field notes on the way teachers conducted circle time activities that, in Spanish nursery schools, are considered as a daily opportunity for promoting communication and group bonding. They are similarly structured in different nursery schools, typically including rounds of presentations, singing, storytelling and/or book-reading, which ensured that all sessions were comparable. Circle time activities were selected because, as structured routines, they account for higher proportions of CDS and could promote more homogeneous and frequent interactions between children and teachers than other school activities (Chaparro-Moreno et al., 2019; Soderstrom et al., 2018).

After the initial observation, the researchers filmed activities by placing two video cameras attached to a tripod, one oriented towards the group of children and the other to the teacher, who also wore a wireless microphone. This ensured that the recording angle and sound allowed for the subsequent identification of their actions. Teachers did not receive any instructions on how to act so as to preserve the ecological conditions of interaction. Overall, we collected over 4 hours of footage in videos with a mean duration of 18.13 min (*SD* = 6.62 min).

### Data Coding

Video files were imported into ELAN, an annotation tool for audio and video recordings (Lausberg & Sloetjes, 2009), where we coded teachers’ communicative bids (i.e. each verbal or gestural attempt to communicate with the children). Teachers’ verbal speech was transcribed according to the CHAT program of the CHILDES project (MacWhinney, 2000), and divided into utterances. Following Bernstein Ratner and Brundage (2015), an utterance or conversational unit is a string of words that ends with a terminal intonation contour, has a complete grammatical structure, or is followed by a pause longer than 2 seconds. We also coded teachers’ communicative bids using predefined categories devised from previous studies (Murillo et al., 2018; Rodrigo et al., 2006). A detailed list of the categories, subcategories, and related examples can be found in Table 1. This included the type of communicative bids, the uses of object and the types of gestures. Additionally, we coded interactive instances according to whether the utterances were addressed to a single child (dyadic) or to the group (polyadic), and also accounted for structural variables such as the classroom layout and the number of children in the classroom. A second researcher independently coded 15% of the recordings (3 out of 16 sessions). Inter-observer agreement ranged from 89% to 99%, indicating a substantial or almost perfect degree of agreement between coders for the use of gestures (89%), the orientation of the communicative bid (89%), and the use of objects (99%). Disagreements between the researchers were discussed to refine the categories for future studies.

**Table 1:** Teachers’ Behavioral Categories Used in This Study

Category / Subcategory	Example
Type of communicative bid	
*Verbal*: Verbal utterances of the teacher.	Posing a question to the group such as “What is the weather like today?”
*Gestural:* Gestures performed by the teacher.	Putting her hand on her head in a gesture of surprise, in response to a child that told her about something that happened to him the day before.
*Verbal-Gestural:* Combined bids of speech and gesture.	Greeting each child by saying “Hello!” while waving her hand.
Uses of object	
*With object:* utterances performed while holding an object and in reference to it.	Showing an object to present it to the group or pointing to a character in a book while shared-reading.
*Without object:* unrelated utterances to an object.	During shared-reading, posing verbal utterances referring to tangentially related topics that were not explicitly pictured in the book.
Type of gestures: Any meaning-loaded motor actions used to communicate with others about a referent or event.	
*Pointing:* gestures that indicate a distal referent. We considered as pointing all gestures performed either with an index finger extension, the whole arm, a head movement or pointing with the chin.	Pointing to her ear and saying to the group "Let's listen to her, please", referring to a girl who is intervening at that moment.
*Instrumental:* gestures performed either to show the object to the interlocutor, to request an object that is out of reach or to transfer the object to someone else. Following Rodrigo et al., (2006) this includes showing, reaching, and giving gestures.	Showing to the children a picture book she chose and seeking their approval before starting to read it.
*Rhythmic:* speech-accompanying gestures that emphasize the rate of language. They generally are harmonic repetitions of the speech utterance.	Clapping to the beat of a song while the children dance around.
*Conventional:* gestures and social routines that are culturally defined.	Nodding to express agreement or waving to say goodbye.
*Symbolic:* gestures that represent absent referents or actions. They have a motivate relationship with what they represent.	With the arms extended, clapping the hands in front of the body in representation of a crocodile’s jaws.
Orientation of the communicative bid	
*Dyadic:* utterances directed to a single child.	Asking a specific child “Who woke you up today?”.
*Polyadic:* utterances directed to the group. They were defined as polyadic if: a) the content of the speech was clearly directed to the group, b) there were vocatives or second person forms in plural, c) teachers’ gaze was directed to the group, and/or d) teachers’ actions referred to the group.	While looking for a book that she wanted to use, the teacher asked the children “Where's the one about walking down the road, kids?”.
Classroom layout	
*Circle:* all children and the teacher were sitting in circle.	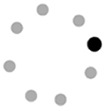
*Line:* the teacher sat in front of the children, who were sitting in line.	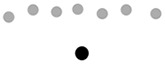
*L-shape:* the teacher was sitting in front of the children, who sat forming an L.	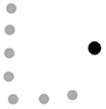
*Undefined:* all children and the teacher sat anywhere in the space, not following a specific order.	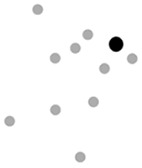

*Note.* For classroom layout examples, we used a dark dot to represent the teacher spatial disposition, while the children are represented in grey color dots.

### Data Analysis

Data wrangling and the calculation of descriptive statistics were performed using SPSS. We analyzed each type of teachers’ utterances by calculating (1) the mean proportion of each type of communicative bid over the total number of communicative bids, and (2) the mean rate per minute (rate/min) of each type of communicative bid. While proportions allow for a general picture of the distribution of each type of bid, their rate/min was considered a better measure due to the different length of the video files. Furthermore, since this dependent variable ensures the independence principle, it was used for comparing the three types of communicative bid (verbal, gestural or a combination of both) through a repeated-measures ANOVA where each type was considered as an intrasubject factor. The same procedure was utilized to explore the distribution of uses of object, of each type of gesture, and whether the utterances were directed to the group or to a single child. In addition, we used Pearson correlations for exploring the relationship between teachers’ uses of object and the different types of gestures, as well as with the number of children that were present in each classroom.

## Results

Across all 16 classrooms, teachers performed 6,575 communicative bids. The most frequent were *verbal* utterances (*n* = 3,486, 53.0%), followed by *verbal-gestural* bids (*n* = 2,911, 44.3%) and *gestures* (*n* = 178, 2.7%). This same distribution applies to their rate/min proportion (Table 2). We found a significant effect of the type of communicative bid, *F*(2,14) = 344.388, *p* < .001, ƞ^2^ = .980. Pairwise comparisons based on the Bonferroni procedure show that there are no significant differences between verbal and verbal-gestural utterances, and that the rate of gestural communicative bids is significantly lower than verbal-gestural (*p* < .001) and verbal productions (*p* < .001). It is worth noting that most teachers’ gesture production did not involve the use of objects, which was only observed in a small portion of the behaviors (*n* = 756, 11.5%).

**Table 2:** Descriptive Statistics for Teachers’ Behaviors

Behavior	Frequency	Rate/min (*M*)	Rate/min (*SD*)
Type of bid			
*Verbal*	3486	12.20	2.95
*Gestural*	178	0.62	0.33
*Verbal-Gestural*	2911	10.62	1.98
Uses of object			
*With object*	756	2.14	2.57
*Without object*	5819	9.88	5.14
Gestures			
*Pointing*	637	2.27	0.69
*Instrumental*	340	1.73	1.67
*Rhythmic*	262	1.03	0.67
*Conventional*	1190	4.58	2.23
*Symbolic*	660	2.39	1.41
Orientation			
*Dyadic*	1148	4.02	1.75
*Polyadic*	1941	0.63	0.18
Classroom layout			
*Circle*	10	n/a	n/a
*Line*	1	n/a	n/a
*L-shape*	4	n/a	n/a
*Undefined*	1	n/a	n/a

Teachers performed unequal numbers of each type of gesture. Of the 3,089 total gestures observed (which include both solely produced gestures and verbal-gestural bids), 1,190 were *conventional* gestures (38.5%), 660 *symbolic* gestures (21.4%), 637 *pointing* gestures (20.6%), 340 *instrumental* gestures (11.0%) and 262 were *rhythmic* gestures (8.5%). We found a significant effect of the type of gesture, *F*(4,12) = 14.959, *p* < .001, ƞ^2^ = .833. Conventional gestures were more frequent than all other types of gestures, with significant differences with pointing (*p* = .014) and rhythmic gestures (*p* < .001). We also found that the differences between conventional and instrumental gestures were marginally significant (*p* = .053), while there was no significance between conventional gestures and the production of symbolic gestures (*p* = .065). Teachers’ use of rhythmic gestures was the least frequent, showing significant differences with pointing (*p* < .001) and symbolic gestures (*p* = .033).

**Figure 1:**
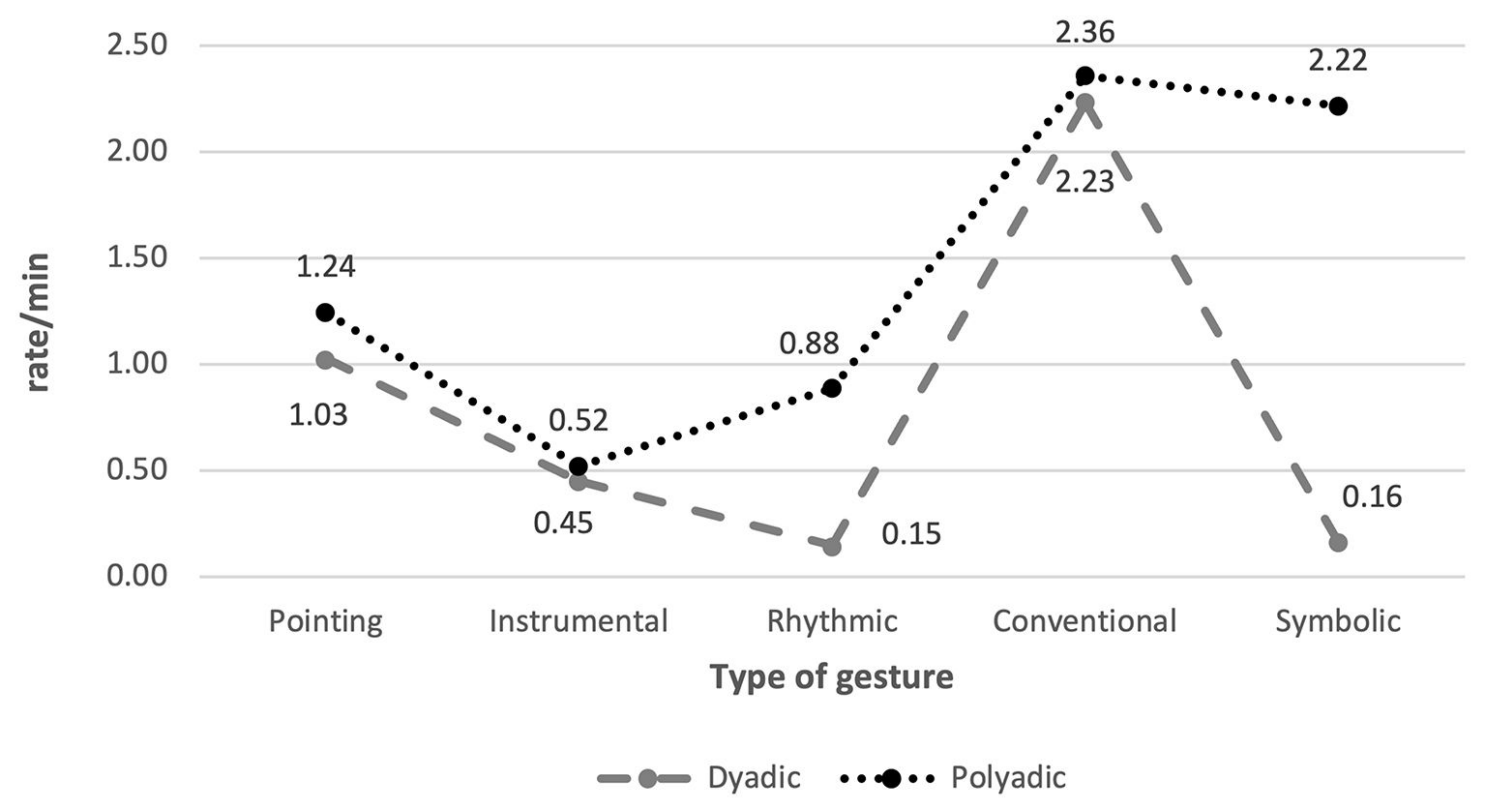
Distribution of Each Type of Gesture According to the Orientation of the Communicative Bids (Dyadic vs. Polyadic)

Interestingly, our data showed that, overall, most bids including gestures were directed to the group (*n* = 1,941, 62.8%), while only around a third of the total gesture production were addressed to a single child (*n* = 1,148, 37.2%). Moreover, the rate/min of dyadic and polyadic gestures significantly differ, *F*(1,15) = 49.733, *p* < .001, ƞ^2^ = .768. Regarding the distribution of each type of gesture, we found a similar trend in gestures addressed to the group and to single children (Figure 1), as evidenced by the effect of the type of gestures with both dyadic, *F*(4,12) = 18.268, *p* < .001, ƞ^2^ = .859, and polyadic instances, *F*(4,12) = 5.061, *p* = .013, ƞ^2^ = .628. This probably results from the higher frequency observed in the production of conventional gestures. However, certain types of gestures were more frequent in polyadic than in dyadic interchanges, as observed for rhythmic, *F*(1,15) = 26.437, *p* < .001, ƞ^2^ = .638, and symbolic gestures, *F*(1,15) = 33.284, *p* < .001, ƞ^2^ = .689.

In order to analyze the relationship between classroom ratios, the type of gesture and the use of objects while gesturing, we ran a series of Pearson bivariate correlations based on the rate/min of each type of gesture and the number of children that were enrolled in each classroom. We found negative and significant correlations between conventional gesture rate/min and the number of children in the classroom, *r*(16) = -.547, *p =* .028. Also, similar results arise from the relationship between gestures and the use of objects, *r*(16) = -.551, *p* = .027.

**Figure 2:**
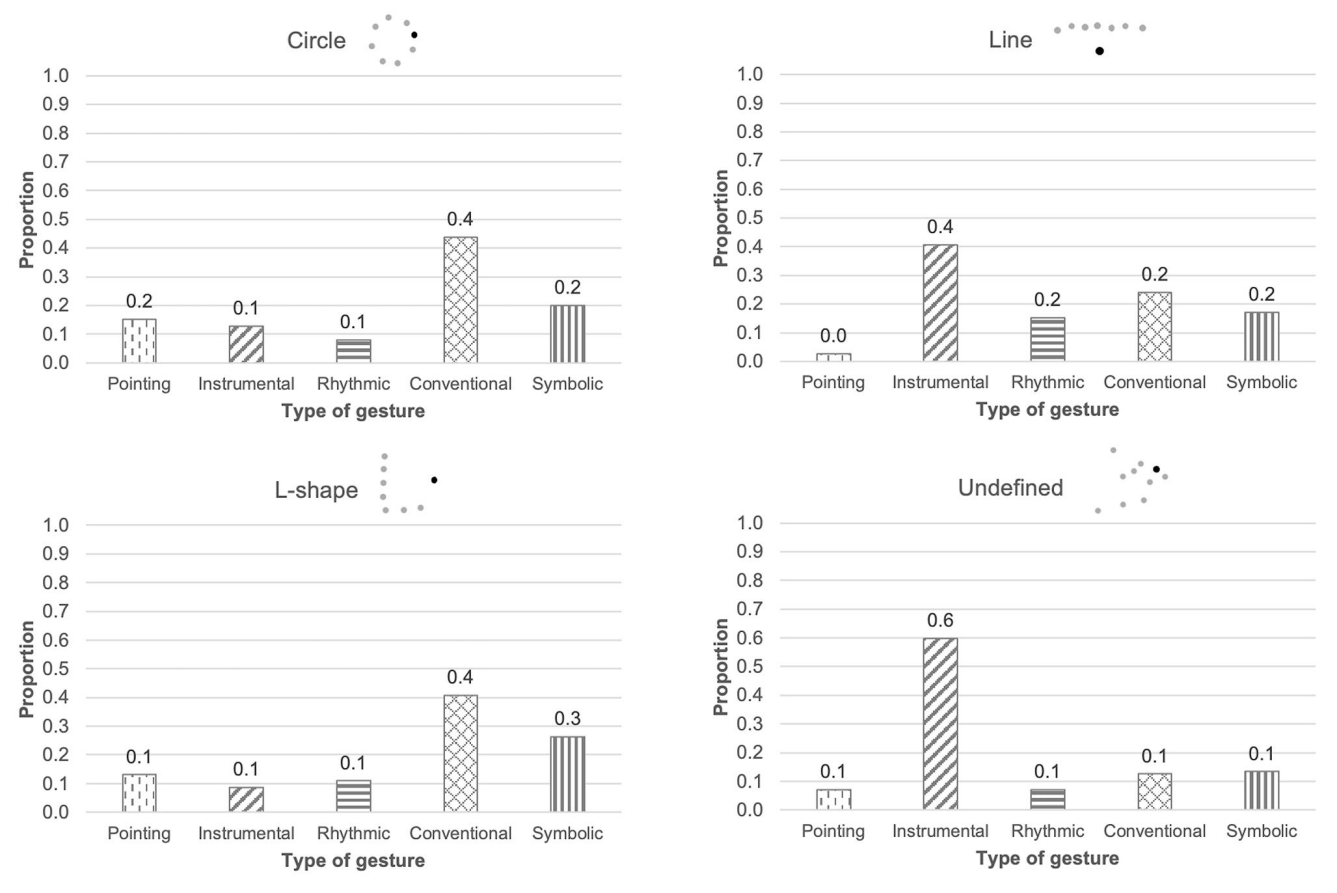
Proportion of Each Type of Gesture According to the Classroom Layout

Finally, in order to explore the relationship between the classroom layout and the type of interaction strategies that teachers promote, we classified each participating classroom according to the participants’ layout (circle, *n* = 10; lines, *n* = 1; L-shape, *n* = 4; undefined, *n* = 1). Our results showed that the use of verbal-gestural communicative bids is similar across all classrooms, except for the classroom with an undefined layout, where the teacher used more verbal utterances (Figure 2). Furthermore, teachers posed unequal uses of gestures with objects, which were very rare when children were sitting in a circle or an L-shape distribution. The distribution of gestures directed to the group and to a single child also differed. Dyadic gestures were more frequent in classrooms where children were arranged in lines or in an L-shape. Note that the classrooms in which children were interacting in a circle or in an undefined layout were the ones that showed the most similar pattern of those observed in the full sample. However, distribution patterns of each type of gesture varied across classroom layouts. While, as shown before, the full sample showed a greater proportion of conventional gestures, we found that this was only observed in those classrooms where children were sitting in a circle or L-shape. In turn, instrumental gestures were more frequent in lineal and undefined layouts where, in fact, this comprised more than 60% of the teachers’ gesture production.

We observed very low frequencies of some types of gestures in some classrooms, that might be related to the spatial organization of the children. For instance, instrumental gestures were the least frequent in L-shaped classrooms, and there was a small representation of pointing gestures in classrooms that were organized in a line or according to an undefined layout (less than 8%). The use of rhythmic gestures was typically low in all classrooms.

## Discussion and Conclusions

The purpose of this study was to examine the relationships between materiality and teachers’ communicative strategies during group interactions in nursery schools with two- to three-year-old children. Our findings show that half of the teachers’ communicative bids during group interactions include the use of gestures, so combined verbal-gestural utterances are more frequent than solely verbal utterances. This is interesting since previous research based on dyadic interactions in home settings showed that only 20% of mothers’ communicative bids include gestures (Casla et al., 2022; Iverson et al., 1999). While adults’ combination of gestures and verbal utterances has been typically seen as an attention getter tool or to direct the child’s action (Iverson et al., 1999; Schaffer et al., 1983; Tamis-LeMonda et al., 2012), it seems that teachers use of these strategies during group interactions ensures an equal distribution of their messages among all children. Additionally, how adults arrange the space and materiality for one-to-one interactions considerably differs from those situations in which a single adult must address her speech to a group of children. Therefore, the specific nature of the relationship between materiality and group interactions in early childhood education contexts needs to be explored.

Overall, we found dissimilar distributions in teacher gesture types, with conventional gestures being more frequent than other types of gestures, such as pointing or instrumental gestures. Previous studies examining mother-child interactions found that pointing gestures were, by far, the most frequent gestures used by mothers (Tamis-LeMonda et al., 2012). In a similar way, mothers use pointing to regulate the child’s attention at 18 months and they are associated with the regulation of the child’s action at 21 months of age (Schmidt, 1996). Moreover, instrumental gestures are also highly frequent in children aged 24 and 36 months (Rodrigo et al., 2006). Surprisingly, neither pointing nor instrumental gestures were the most frequently observed in our data, which could partially explain why the proportion of gestures with objects was significantly lower than those of gestures without objects. While instrumental gestures usually involve transferring an object from the hands of one communicative partner to another, circle time activities in our sample were not typically object-centered. Instead, these activities are usually devoted to conversational group interchanges or to the participation of all children and teachers in rhymes, routines and songs. This may need more attention-getting strategies, such as conventional gestures, than action regulation. In this sense, conducting longitudinal studies and ensuring a diverse sample in terms of type of providers, SES background and children ages could shed light on whether the use of objects is typically scarce in this type of group interactions, or whether it varies according to different contexts and/or moments over the early years’ education (Hansen, 2018).

As normalized forms of conveying information, conventional gestures are more easily addressed to the whole group. Indeed, the negative correlation between both the use of conventional and instrumental gestures and the number of children suggests that gestures with objects might be more frequent in smaller group interactions, as they allow for greater individual participation (Degotardi, 2021). Also, the fact that the use of objects was very infrequent in approximately 40% of the classrooms in our study does not necessarily mean that educators in these classrooms neglect the importance of materiality. Perhaps they simply did not use objects during circle time on that particular day, which could be due to a variety of reasons (e.g., because the children’s conversation was fluent enough not to require extra material support, or because they tend to use objects in different routines/moments). Follow-up studies should investigate the reasons behind limited use of objects in certain classrooms and if it could be associated with structural factors.

Moreover, we found dissimilar distributions in teacher gesture type used for addressing the group or a single child during circle time interactions. Although, as mentioned before, conventional gestures were the most frequent in both cases, it is interesting that symbolic and rhythmic gestures, specific to songs and rhymes, were more frequently addressed to the group than to individual children. The differences between dyadic and polyadic gestures highlight the importance of considering the specific classroom layout during interactions, as they may promote diverse communicative behaviors from children (Løkken & Moser, 2012). Thus, it could be interesting to investigate if, while engaging many children in the interactions, teachers are aligned with specific features of quality teacher-child interactions (Pianta, 2016), such as encouraging children to brainstorm their own ideas, acknowledging their interventions by repeating or rephrasing their responses (Casla et al., 2022), and providing positive and encouraging feedback.

Even when the overall proportion of conventional gestures was very high, we observed how the specific proportion of each type of gesture differed across the various classroom layouts. Particularly, when children were sitting in lines or moving around the classroom (i.e., in an undefined layout), teachers seem to promote more one-to-one exchanges than in other spatial distributions. Considering that, in essence, these two layouts are quite different from each other, we should delve deeper into the reasons that could underlie these similarities. First, lineal layouts do not facilitate peer interaction as children cannot see most of their classmates, and thus interaction and attention became teacher-centered. Conversely, in undefined layouts children often stood up to get close to the teacher, showing her objects or trying to get her attention (e.g., poking her shoulder), which resulted in increased child-teacher direct interactions. Also, the use of instrumental gestures reached 60% in both lineal and undefined layouts, thus reaching a similar proportion to that observed in dyadic interactions at home (Rodrigo et al., 2006). While classroom group interactions are evidently not fully comparable with mother-child dyadic interaction because, for instance, in the classroom children also share interactions with their peers, our results indicate that circle time could be a rich interactive environment if it also includes one-to-one interchanges of objects and speech (Zahavi, 2021, 2022).

Though our goal was not to make generalizable claims about the educators’ speech in the early years, this study is the first step to depict the specific relationships between materiality and multimodal communication beyond mother-child dyadic interaction. Further research should increase the sample size and its diversity to yield more representative results. In particular, while most classrooms conduct circle time activities and have a reasonable variety of materials available, we need to know whether they are also accessible for children, i.e., if they are given opportunities for exploration and free-choice (Bautista et al., 2020). Limited accessibility to certain spaces and/or certain resources/materials without the supervision of the educator could lead to substantial differences in the type of interaction that is promoted in the classroom. Moreover, future studies should analyze children’s responses to the communicative bids that teachers use (Cremin et al., 2017) and explore similarities and differences across the various classroom layouts. This could potentially raise awareness for early childhood practitioners about the importance of enacting more constructive forms of interactions with a group of young children. Likewise, the scientific research on early childhood education could be enriched by adopting a longitudinal perspective that, in turn, would inform the analysis of adult mediation in early child development and its role in fostering opportunities for children’s participation. This would allow for a comprehensive analysis of the origin and changing nature of the communicative exchanges between educators, toddlers and materiality, as well as the importance of spaces in promoting or restricting certain classroom interactions. While circle time may include various types of activities (e.g. presentations, book reading, storytelling, singing), its relationship with the nature of communicative behaviors is still underexplored. As such, mapping the frequency and proportion of time dedicated to each of those activities may contribute to better depicting how circle time interactions unfold.

All in all, our results show that teachers’ communication with toddlers is highly multimodal and could be shaped according to the interactive opportunities and limitations that the classroom layout offers. Even when circle time is defined as a group activity that promotes linguistic socialization (Poveda, 2003) and that fosters opportunities for conversational interchanges and the production of linguistic utterances (Chaparro-Moreno et al., 2019), we need to know better how they are mediated by the material and spatial organization. Despite the limitations of this study, results suggest that there is a manifest distinction between dyadic interactions at home and group (polyadic) interactions in the nursery school context, including differences in the use of objects and space. These differences should be further explored to understand the role of other factors (such as the school context, the children ages, or the nature of the observed activities) in the opportunities and limitations that teachers may foster through their interactive proposals. It is worth noting that most research on circle time interactions has been conducted in kindergartens with children over three years of age. Therefore, this study is significant because it also enriches the limited classroom-based research literature with younger children by providing new data on classroom interactions from a non-English-speaking country. Our study proposes exploring a topic with educational and psychological interest for early infancy, given that group classroom activities have demonstrated a wide potential to actively involve children and encourage their participation in social interactions.

## References

[r1] Alessandroni, N. (2023). The road to conventional tool use: Developmental changes in children’s material engagement with artifacts in nursery school. Infancy, 28(2), 388–409. 10.1111/infa.12522 36571567

[r2] Bautista, A., Moreno-Núñez, A., Vijayakumar, P., Quek, E., & Bull, R. (2020). Gross motor teaching in preschool education: Where, what, and how do Singapore educators teach? Infancia y Aprendizaje, 43(2), 443–482. 10.1080/02103702.2019.1653057

[r3] Berge, A. (2019). ‘I believe that a sofa can be of great help’: Materiality in a kindergarten room and the impact on social practices. International Journal of Early Childhood, 51(1), 59–72. 10.1007/s13158-019-00235-6

[r4] Bernstein Ratner, N., & Brundage, S. (2015). *A clinician’s complete guide to CLAN and PRAAT.* Talkbank Organization. https://talkbank.org/manuals/Clin-CLAN.pdf

[r5] Cárdenas, K., Moreno-Núñez, A., & Miranda-Zapata, E. (2020). Shared book-reading in early childhood education: Teachers’ mediation in children’s communicative development. Frontiers in Psychology, 11, 2030. 10.3389/fpsyg.2020.02030 33013511 PMC7505735

[r6] Casla, M., Méndez-Cabezas, C., Montero, I., Murillo, E., Nieva, S., & Rodríguez, J. (2022). Spontaneous verbal repetition in toddler-adult conversations: A longitudinal study with Spanish-speaking two-year-olds. Journal of Child Language, 49(2), 266–301. 10.1017/S0305000921000015 33736727

[r7] Chaparro-Moreno, L. J., Justice, L. M., Logan, J. A. R., Purtell, K. M., & Lin, T. J. (2019). The preschool classroom linguistic environment: Children’s first person experiences. PLoS One, 14(8), e0220227. 10.1371/journal.pone.0220227 31390357 PMC6685670

[r8] Cremin, T., Flewitt, R., Mardell, B., & Swann, J. (2017). *Storytelling in early childhood: Enriching language, literacy, and classroom culture*. Routledge.

[r9] Dalgaard, N. T., Bondebjerg, A., Klokker, R., Viinholt, B. C. A., & Dietrichson, J. (2022). Adult/child ratio and group size in early childhood education or care to promote the development of children aged 0–5 years: A systematic review. Campbell Systematic Reviews, 18(2), e1239. 10.1002/cl2.1239 36911342 PMC9066244

[r10] Degotardi, S. (2021). The language environment of infant child care: Issues of quantity, quality, participation, and context. In O. Saracho (Ed.), *Contemporary perspectives on research on child care in early childhood education* (pp. 85–107). Information Age Publishing.

[r11] Degotardi, S., Han, F., & Torr, J. (2018). Infants’ experience with ‘near and clear’ educator talk: Individual variation and its relationship to indicators of quality. International Journal of Early Years Education, 26(3), 278–294. 10.1080/09669760.2018.1479632

[r12] García, J. L., Heckman, J. J., Leaf, D. E., & Prados, M. J. (2020). Quantifying the life-cycle benefits of an influential early-childhood program. Journal of Political Economy, 128(7), 2502–2541. 10.1086/705718 32616965 PMC7331936

[r13] Hansen, J. E. (2018). *Educational language practices and language development in early childhood education and care* [Doctoral dissertation, University of Stavanger]. https://hdl.handle.net/11250/2712081

[r14] Hindman, A. H., Farrow, J. M., Anderson, K., Wasik, B. A., & Snyder, P. A. (2021). Understanding child-directed speech around book reading in toddler classrooms: Evidence from Early Head Start programs. Frontiers in Psychology, 12, 719783. 10.3389/fpsyg.2021.719783 34955952 PMC8695438

[r15] Iverson, J. M., Capirci, O., Longobardi, E., & Caselli, M. C. (1999). Gesturing in mother-child interactions. Cognitive Development, 14(1), 57–75. 10.1016/S0885-2014(99)80018-5Iverson

[r16] Justice, L. M., Jiang, H., & Strasser, K. (2018). Linguistic environment of preschool classrooms: What dimensions support children’s language growth? Early Childhood Research Quarterly, 42(1), 79–92. 10.1016/j.ecresq.2017.09.003

[r17] Kärtner, J. (2018). Beyond dichotomies—(M)others’ structuring and the development of toddlers’ prosocial behavior across cultures. Current Opinion in Psychology, 20, 6–10. 10.1016/j.copsyc.2017.07.040 28822898

[r19] Lausberg, H., & Sloetjes, H. (2009). Coding gestural behavior with the NEUROGES-ELAN system. Behavior Research Methods, 41(3), 841–849. 10.3758/BRM.41.3.841 19587200

[r20] Løkken, G., & Moser, T. (2012). Space and materiality in early childhood pedagogy – Introductory notes. Education Inquiry, 3(3), 303–315. 10.3402/edui.v3i3.22036

[r21] MacWhinney, B. (2000). *El Proyecto CHILDES: Herramientas para el análisis del habla.* Lawrence Erlbaum.

[r22] Moreno-Núñez, A., Rodríguez, C., & del Olmo, M. J. (2017). Rhythmic ostensive gestures: How adults facilitate babies’ entrance into early triadic interactions. Infant Behavior and Development, 49, 168–181. 10.1016/j.infbeh.2017.09.003 28946022

[r23] Murillo, E., Ortega, C., Otones, A., Rujas, I., & Casla, M. (2018). Changes in the synchrony of multimodal communication in early language development. Journal of Speech, Language, and Hearing Research: JSLHR, 61(9), 2235–2245. 10.1044/2018_JSLHR-L-17-0402 30090947

[r24] Perniss, P. (2018). Why we should study multimodal language. Frontiers in Psychology, 9, 1109. 10.3389/fpsyg.2018.01109 30002643 PMC6032889

[r25] Perry, L. K., Prince, E. B., Valtierra, A. M., Rivero-Fernández, C., Ullery, M. A., Katz, L. F., Laursen, B., & Messinger, D. S. (2018). A year in words: The dynamics and consequences of language experiences in an intervention classroom. PLoS One, 13(7), e0199893. 10.1371/journal.pone.0199893 29979740 PMC6034821

[r26] Pianta, R. C. (2016). Teacher-student interactions: Measurement, impacts, improvement, and policy. Policy Insights from the Behavioral and Brain Sciences, 3(1), 98–105. 10.1177/2372732215622457

[r27] Poveda, D. (2003). Paths to participation in classroom conversations. Linguistics and Education, 14(1), 69–98. 10.1016/S0898-5898(03)00007-X

[r28] Rodrigo, M. J., González, A., Ato, M., Rodríguez, G., de Vega, M., & Muñetón, M. (2006). Co-development of child–mother gestures over the second and the third years. Infant and Child Development, 15(1), 1–17. 10.1002/icd.412

[r29] Rowe, M. L., & Goldin-Meadow, S. (2009). Early gesture selectively predicts later language learning. Developmental Science, 12(1), 182–187. 10.1111/j.1467-7687.2008.00764.x 19120426 PMC2677374

[r30] Rowe, M. L., & Snow, C. E. (2020). Analyzing input quality along three dimensions: Interactive, linguistic, and conceptual. Journal of Child Language, 47(1), 5–21. 10.1017/S0305000919000655 31668157

[r31] Schaffer, H. R., Hepburn, A., & Collis, G. M. (1983). Verbal and nonverbal aspects of mothers’ directives. Journal of Child Language, 10(2), 337–355. 10.1017/S0305000900007807 6874771

[r32] Schmidt, C. L. (1996). Scrutinizing reference: How gesture and speech are coordinated in mother-child interaction. Journal of Child Language, 23(2), 279–305. 10.1017/S0305000900008801 8936688

[r33] Soderstrom, M., Grauer, E., Dufault, B., & McDivitt, K. (2018). Influences of number of adults and adult: Child ratios on the quantity of adult language input across childcare settings. First Language, 38(6), 563–581. 10.1177/0142723718785013

[r34] Soderstrom, M., & Wittebolle, K. (2013). When do caregivers talk? The influence of activity and time of day on caregiver speech and child vocalizations in two childcare environments. PLoS One, 8(11), 80646. 10.1371/journal.pone.0080646 24260443 PMC3832484

[r35] Suanda, S. H., Smith, L. B., & Yu, C. (2016). The multisensory nature of verbal discourse in parent–toddler interactions. Developmental Neuropsychology, 41(5-8), 324–341. 10.1080/87565641.2016.1256403 28128992 PMC7263485

[r36] Tamis-LeMonda, C., Song, L., Smith Leavell, A., Kahana-Kalman, R., & Yoshikawa, H. (2012). Ethnic differences in mother–infant language and gestural communications are associated with specific skills in infants. Developmental Science, 15(3), 384–397. 10.1111/j.1467-7687.2012.01136.x 22490178

[r37] Torr, J. (2019). Infants’ experiences of shared reading with their educators in early childhood education and care centres: An observational study. Early Childhood Education Journal, 47(5), 519–529. 10.1007/s10643-019-00948-2

[r38] Vermeer, H. J., van Ijzendoorn, M. H., Cárcamo, R. A., & Harrison, L. J. (2016). Quality of child care using the environment rating scales: A meta-analysis of international studies. International Journal of Early Childhood, 48(1), 33–60. 10.1007/s13158-015-0154-9

[r39] White, E. J., Peter, M., & Redder, B. (2015). Infant and teacher dialogue in education and care: A pedagogical imperative. Early Childhood Research Quarterly, 30, 160–173. 10.1016/j.ecresq.2014.10.008

[r40] Wu, Z., & Gros-Louis, J. (2014). Infants’ prelinguistic communicative acts and maternal responses: Relations to linguistic development. First Language, 34(1), 72–90. 10.1177/0142723714521925

[r41] Zahavi, D. (2021). We in me or me in we? Collective intentionality and selfhood. Journal of Social Ontology, 7(1), 1–20. 10.1515/jso-2020-0076

[r42] Zahavi, D. (2022). Individuality and community: The limits of social constructivism. Ethos: Journal of the Society for Psychological Anthropology, 50(4), 392–409. 10.1111/etho.12364 PMC10099490 37064549

